# Establishment of monoclonal HCC cell lines with organ site-specific tropisms

**DOI:** 10.1186/s12885-015-1692-0

**Published:** 2015-10-12

**Authors:** Jinliang Wan, Duo Wen, Lili Dong, Jun Tang, Dongli Liu, Yang Liu, Zhonghua Tao, Dongmei Gao, Huichuan Sun, Ya Cao, Jia Fan, Weizhong Wu

**Affiliations:** 1 Liver Cancer Institute and Zhongshan Hospital, Fudan University, Key Laboratory of Carcinogenesis and Cancer Invasion, Ministry of Education, Shanghai, 200032 China; 2 Department of Oncology, Affiliated Hospital of Binzhou Medical University, Binzhou, Shandong Province 256603 People’s Republic of China; 3 Institute of Biomedical Sciences of Fudan University, Shanghai, 200032 China; 4 Cancer Research Institute, Key Laboratory of Carcinogenesis and Cancer Invasion, Ministry of Education, Central South University, Changsha, Hunan China; 5 Liver Cancer Institute and Zhongshan Hospital, Fudan University, 180 Fenglin Road, Shanghai, 200032 China

**Keywords:** Hepatocellular carcinoma, Monoclonal cell line, Metastasis, Organ-site tropism, Xenograft model

## Abstract

**Background:**

Organ site-specific metastasis is an ominous feature for most poor-prognostic hepatocellular carcinoma (HCC) patients. Cancer cell lines and animal models are indispensable for investigating the molecular mechanisms of organ specific tropism. However, till now, little is known about the drivers in HCC metastatic tropism, and also no effective way has been developed to block the process of tropistic metastasis.

**Methods:**

In this study, we established several monoclonal HCC cell lines from HCCLM3-RFP together with their xenograft models, and then analyzed their metastatic potentials and tropisms using in-vitro and in-vivo assays, and finally elucidated the driving forces of HCC tropistic metastases.

**Results:**

Six monoclonal cell lines with different organ site-specific tropism were established successfully. SPARC, VCAM1 and ANGPTL4 were found positively correlated with the potentials of lung metastasis, while ITGA1 had a positive relation to lymph node metastasis of enterocoelia.

**Conclusions:**

By our powerful platforms, HCC metastatic tropisms in clinic could be easily mimicked and recapitulated for exploring the bilateral interactions between tumor and its microenvironment, elucidating the drivers of HCC metastatic tropisms, and testing anti-cancer effects of newly developed agent in pre-clinical stage.

**Electronic supplementary material:**

The online version of this article (doi:10.1186/s12885-015-1692-0) contains supplementary material, which is available to authorized users.

## Background

Hepatocellular carcinoma (HCC) is the third common cause of cancer death worldwide [1]. Tumor metastasis to distant vital organs is responsible for more than 90 % HCC-related fatalities although the metastatic process is claimed to be an inefficient on [2, 3]. In the process, the vast majority of circulating tumor cells is incapable of progressive metastasis growth at distant organs [4, 5]. Furthermore, tumor metastasis often exhibit organ-site specific tropism, preferring to grow in certain organ [6, 7]. For example, HCC is more likely to metastasize to lung, lymph node and adrenal gland, but less frequently to bone and brain. Although the phenomena have been observed for several decades in clinic, their molecular bases on HCC metastatic tropism are still poorly understood.

Tumor metastasis is a multiple complex process which is driven by an accumulation of genetic mutations and detuning expression, making a few tumor cells an acquisition of a full ability of organ-site specific metastasis during its evolutional process [8]. Based on our previous knowledge, it is easy to understand that the community of cancer cells is a collection of heterozygosis [9]. In fact, several established breast cancer and prostate cancer cell lines with bone and/or lymph node metastatic tropisms support the idea of cancerous heterogeneity. Up to now, however, no such cell lines and animal models are available for studying the metastatic tropisms of HCC. Little is known about the drivers in HCC metastatic tropism, and also no effective way has been developed to block the process of tropistic metastasis. Therefore, it is desperately needed to develop several monoclonal HCC cell lines with different organ-site tropisms to identify the underlying mechanisms of this phenotype.

In the past decade, researchers from Liver Cancer Institute of Fudan University have successfully established two human HCC xenografts in nude mice, namely LCI-D20 and LCI-D35, via orthotopic transplantation of tumor tissues from HCC patients, and then successfully cloned several HCC cell lines with different metastatic potentials from LCI-D20 xenografts, such as MHCC97, HCCLM3 and HCCLM6 [10–12]. More recently, our group developed a red fluorescent protein-expressing HCC cell line, HCCLM3-RFP (HCCLM3-R), whose xenograft models in nude mice can provide a real-time observation of tumor growth and metastasis [13–15]. In this study, we intended to establish several monoclonal HCC cell lines from HCCLM3-RFP, and then analyze its metastatic potentials and tropisms, and finally elucidate the driving forces of HCC tropistic metastases.

## Methods

### Animals

Male BALB/c nu/nu mice with 4-6 weeks old were obtained from the Shanghai Institute of Materia Medica, Chinese Academy of Science (CAS), China, and housed in laminar-flow cabinets under specific pathogen-free conditions. The mice were kept for 5-7 days as an adaptation period before being subjected to experimental use. The study protocol on mice was approved by the Shanghai Medical Experimental Animal Care Committee. All animals received humane care according to the criteria outlined in the "Guide for the Care and Use of Laboratory Animals" prepared by the National Academy of Sciences and published by the National Institutes of Health (NIH publication 86-23 revised 1985).

### Cell culture

HCCLM3 was established by Dr. Li et al. in our institute [11]. HCCLM3-R with high metastatic potential, used as parental cell line in the study, was maintained in high-glucose Dulbecco’s modified eagle medium (D-MEM; GibcoBRL, Grand Island, NY) supplemented with 10 % fetal bovine serum (GibcoBRL, Grand Island, NY) in a humidified atmosphere of 5 % CO_2_ at 37 °C.

### Human ethics

Human ethics approval was not required for any aspect of this study.

### Establishment of HCCLM3-RFP xenograft models

Male athymic BALB/c nude mice with 4 weeks old (Institute of Materia, CAS, Shanghai, China) were injected each with 1 × 10^7^ HCCLM3-R cells in 200 μl 0.9 % sodium chloride solution on the right upper flank region to establish subcutaneous xenograft model. When it reached 1 cm in diameter 3 or 4 weeks later, the subcutaneous tumor was removed, cut into pieces in a size of 2 × 2 × 2 mm^3^ and implanted into the livers of another 8 nude mice, as described previously [11].

### Colonization of single-cell derived HCC population

By virtue of RFP signaling, metastatic tissues were biopsied directly from the lung and lymph node of orthotropic HCCLM3-R xenograft models. The first descendant cells from lung and lymph node metastatic foci, namely LM-1 and LnM-1 respectively, were trypsinized, washed with PBS, and resuspended at 1000 cells/ml. The suspensions were serially diluted in 10-fold volume of fresh media. One hundreds μl of such diluted suspension was added into the well of 96-well plate. After 24 h, these wells harboring a single cell were identified and labeled under microscope. Two weeks later, only colonies derived from one single cell were trypsinized and sub-cultured gradually using a 12-well plate, 6-well plate, and then a 15 cm tissue flask.

### Metastatic tropism of monoclonal HCC cells

Each monoclonal HCC cell line was used to establish xenograft models in 6 nude mice. On the 6th week after orthotopic transplantation, all animals were sacrificed for autopsy. Spontaneous metastasis to liver, lung and celiac lymph nodes were imaged under stereomicroscope (Leica, Heerbrugg, Switzerland). The integrated optical density (IOD) of the tumor fluorescence was quantitated by Image Pro Plus software 6.0 (Media Cybernetics, Bethesda, MD). And the metastatic tropism of each monoclonal HCC cell line was plotted using IOD.

### Pathological examination

Orthotopic tumor and its metastatic foci were resected for histopathological studies. After fixed with 4 % formaldehyde, lung metastasis tissue were embedded in paraffin and serially cut for hemotoxylin and eosin staining. Tumor metastatic potential in each monoclonal HCC xenograft was analyzed by the following semi-quantitative scoring system as described by Dr. Gao [16], which is advantage to evaluate tumor metastatic potential, even when the metastatic foci in a given organ are overlapped into a cluster. In detail, Grade I: the diameter of metastatic foci in lung were less than 0.5 mm; Grade II: the diameter of foci was between 0.5 and 1 mm; Grade III: the diameter of foci was between 1 and 2 mm; Grade IV: the diameter of foci was more than 2 mm. For a large metastatic tumor, the number of foci was multiple of grade IV. Then, the total metastatic foci number of each monoclonal HCC cell line = grade I × 1+ grade II × 2+ grade III × 3+ grade IV × 4.

### Lung colonization assays

A tail vein injection model was employed for lung colonization assays [16]. In brief, nude mice were injected with 1 × 10^5^ of viable parent and monoclonal HCC cells via a lateral tail vein. Three days later, the lungs were removed for making continuous frozen section. The colonized foci of HCCLM3-R and its derived monoclonal cells in lung were quantified by fluorescence microscope.

### Cell proliferation assays

Cell proliferation was determined by the Cell Counting Kit-8 assay (Dojin Laboratories, Kumamoto, Japan). In brief, HCCLM3-R and its derived monoclonal cells in exponential growth phase were trypsinized to give single-cell suspension. Then the cell concentration was adjusted into 2 × 10^4^ /ml, and added 100 μl of cell suspension into each well of a 96-well plate. After a cell culture of 0-, 24-, 48-, 72- and 96-h at 37 °C with 5 % of CO_2_, 10 μl of the kit reagent was added to each well. Two hours later, all plates were scanned by a microplate reader (Thermo Fisher Scientific, Waltham, MA) at 450 nm. Cell proliferation was calculated on the basis of absorbency.

### Migration and invasion assays

Cell migration and invasion was analyzed by a Transwell™ Permeable Supports system (Corning, Corning, NY) according to the manufacturer’s instruction. HCCLM3-R and its derived monoclonal cells were seeded into uncoated upper insert at 5 × 10^4^ cells for migration assay and seeded into a Matrigel (BD Biosciences, San Jose, CA) coated upper insert at 1 × 10^5^ cells for invasion assay. Then, the medium containing 10 % fetal bovine serum as a chemoattractant was added into the lower well. Following a culture of 24 or 48 h, non-invading cells were removed from the upper surface by wiping with a cotton swab. The membrane was fixed with 4 % formaldehyde for 15 min at room temperature. The invading cells were stained with Giemsa (Sigma, St Louis, MO) for 25 min, and its numbers in ten fields of each triplicate filter were analyzed by inverted microscope.

### RNA extraction and real-time PCR

Total RNA was purified using TRIzol Reagent (Invitrogen Life Technologies, Carlsbad, CA) following the manufacturer’s instruction. The quality of RNA was examined by A260 absorption. And 500 ng of total RNA was reversely transcribed into first-strand cDNA using PrimeScript RT reagent kit (Takara, Tokyo, Japan). Real-time PCR in triplicate was performed on a DNA Engine Opticon system (MJ Research, Reno, NV) using SYBR Premix Ex Taq (TaKaRa, Tokyo, Japan). And the reaction was evaluated with the Opticon Monitor software (Version 1.02). The primer sequences for interested mRNAs were synthesized by Sangon Biotech (Sangon Biotech, Shanghai, China; Additional file 1: Table S1). The threshold cycle (Ct) values were analyzed using the comparative Ct (ΔΔCt) method as previously reported [17]. The level of target mRNA was obtained by normalizing to the endogenous reference and relative to a control.

### Western blot analysis

Total cell protein was extracted from HCCLM3-R and its derived monoclonal cells with ProteoJETTM Mammalian Cell Lysis Reagent (Fermentas, Waltham, MA), supplemented with phenylmethanesulfonyl fluoride (PMSF; Roche, Indianapolis, IN). Equal protein amount was loaded onto 10 % SDS-PAGE gels. The protein samples were separated and transferred onto PVDF membranes (Millipore, Billerica, MA) for 2.5 h at 110 V. After blocked with 5 % milk solution in TBS for 1 h at room temperature, the membrane was incubated with rabbit antibodies against VCAM1 (1:1000, Epitomics, Burlingame, CA), SPARC (1:1000, Epitomics, Burlingame, CA), ANGPTL4 (1:1000, Abgent, San Diego, CA) and ITGA1 (1:1000, Abgent, San Diego, CA) overnight at 4 °C respectively. Membranes were washed extensively and then incubated for 1 h with anti-rabbit IgG conjugated to HRP (1:5000). The protein bands were visualized using enhanced chemiluminescence reagents (Thermo Fisher Scientific, Waltham, MA), acquired by Molecular Imager ChemiDox XRS + Imaging System (Bio-Rad Laboratories, Hercules, CA) and quantified with Gel-Pro Analyzer (United Bio, Sanborn, NY).

### Immunohistochemistry assays

Immunohistochemistry for target molecules was performed on single serial section made from primary tumor samples. The slides probed with a primary antibody against VCAM1 (1:200, Epitomics, Burlingame, CA), SPARC (1:200, Epitomics, Burlingame, CA), ANGPTL4 (1:100, Abgent, San Diego, CA) and ITGA1 (1:100, Abgent, San Diego, CA), and then incubated with HRP (horseradish peroxidase) -conjugated IgG (1:500, Invitrogen) and the proteins in situ were visualized with 3, 3’-diaminobenzidine. Density of target proteins was determined as previous report [18].

### Statistical analysis

All values are expressed as mean ± standard deviation. Data were analyzed by the computer program Graphpad Prism 5 software [16]. The number of lung metastasis in histopathological examinations, were shown by median. Quantitative variables were analyzed by One-way ANOVA, Kruskal-Wallis test or Fisher exact test to compare the qualitative variables. Other comparisons were performed by using unpaired 2-sided Student’s *t* test without equal variance assumption or nonparametric Mann-Whitney test. Results were considered statistically significant at p < 0.05.

## Results

### First descendent cell lines from HCCLM3-R with organ tropism

**Fig. 1:**
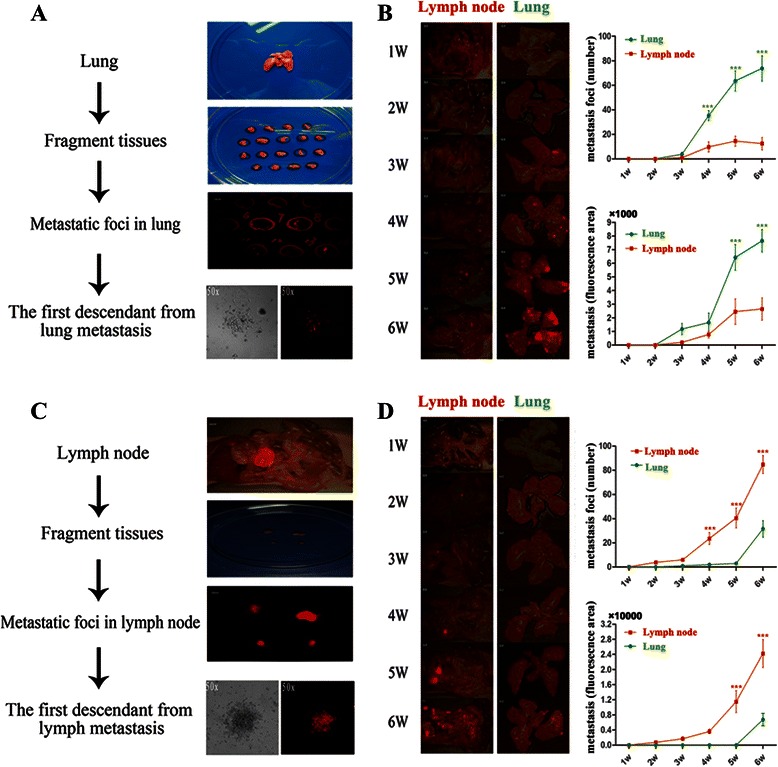
Schematical diagrams of LM-1 and LnM-1 establishments and their metastatic tropisms. **a** & **c** Establishing approaches of LM-1 and LnM-1 cells from HCCLM3-R xenograft models. **b** & **d** Representative images of HCC metastatic patterns in LM-1 and LnM-1 orthotropic xenograft models (*left*). Quantitative analyses of HCC metastases in lung and lymph node with foci number as well as fluorescence area (*right*). Data are presented as mean ± SEM, Wilcoxon rank testing was used

As shown in Fig. 1a and c, metastatic tissues with RFP signaling were biopsied directly from lung and lymph node of orthotropic HCCLM3-R xenograft models. The first descendant cells from lung and lymph node metastatic foci were named LM-1 and LnM-1, respectively. The lineages of HCCLM3-R and its derived clones were examined by exon sequencing. And a similar LOH expression pattern was found in these 3 cells lines, indicating that the derived 2 clones came from the same parental cell line (data unshown). To investigate the organ-specific tropisms of these two cell lines, subcutaneous and orthotropic xenograft models were built in nude mice. Indeed, LM-1 xenografts, during a 6-weeks observation, apparently displayed more lung metastatic capabilities than lymph node (Fig. 1b). As anticipated, LnM-1 showed significantly metastatic tropism to lymph node than lung metastases (Fig. 1d). A district metastatic propensity curves was obtained between LM-1 and LnM-1 cell lines (Fig. 1b and d). Therefore, the capacities of organ-specific metastasis were able to be inherited in its descendent cells.

### Metastatic tropism of single cell–derived populations from HCCLM3-R

**Fig. 2:**
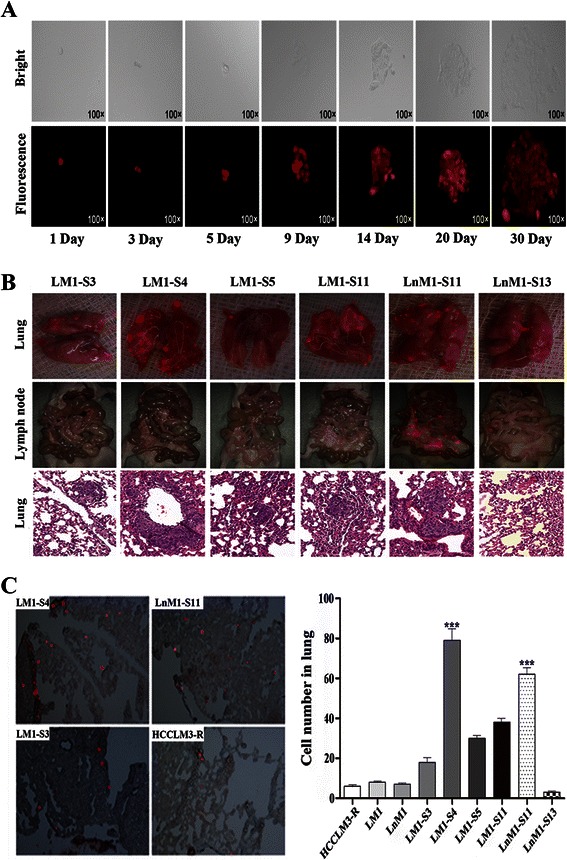
Metastatic tropisms and lung colonizing abilities of monoclonal HCC cell lines. **a** Representative images of the screening processes of monoclonal cell line under bright field (*top*) and red fluorescence field (*bottom*, ×100). **b** Metastatic foci in lung (*top*, ×1.25) and celiac lymph node (*middle*, ×0.63) visualized via fluorescence microscope; lung metastasis (*bottom*, ×200) verified in H&E-stained sections. **c** Lung colonies of monoclonal HCC cells in the recipient mice at 72 h after tail vein injection (*left*); Quantification of colonizing cells in frozen sections of lung (*right*). Data are presented as mean ± SEM, Kruskal-Wallis was used

In order to establish monoclonal HCC cell lines with organ specific metastatic potentials, a total of 155 single-cell wells were obtained by limiting dilution of LM1 and LnM1 cells (Fig. 2a). Among them, only 13 subclones grew well in vitro system. All of them displayed an epithelioid, polygonal-shaped morphology, which were similar to their parental cell line, HCCLM3-R. Six of thirteen (6/13) monoclones, named LM1-S3, LM1-S4, LM1-S5, LM1-S11, LnM1-S11 and LnM1-S13, were successfully established subcutaneous and then orthotopic xenograft models. The representative metastatic patterns of these 6 monoclones were imaged by fluorescence microscope (Fig. 2b), quantified by IOD assay and pathological examination after a 6-weeks implantation (Fig. 2b). Our results showed once more that distinct metastatic abilities of 6 monoclonal cell lines were displayed in both of metastatic inclination and metastatic potential. Lung metastatic tropisms were found in LM1-S3, LM1-S4, LM1-S5 and LM1-S11 derived xenograft models, And, LM1-S4 had the highest potential of lung metastasis among these monoclonal cell lines (*p* <0.01; Table 1). More interesting, LnM1-S11 exhibited dual organ metastatic potentials, both to lung and celiac lymph nodes (*p* < 0.05; Table 2). In our histopathological examinations, the median of lung metastases (Q2) in LM1-S3, LM1-S4, LM1-S5, LM1-S11, LnM1-S11 and LnM1-S13 xenograft models were 102 (Q1 = 99, Q3 = 121), 1426 (Q1 = 1230, Q3 = 1655), 70 (Q1 = 59, Q3 = 85), 621 (Q1 = 588, Q3 = 694), 564 (Q1 = 535, Q3 = 611) and 477 (Q1 = 420, Q3 = 526) respectively. These results were further confirmed by IOD quantitative study (Table 2).

**Table 1:** Incidence rate of lung and lymph node metastases of monoclonal HCC cell lines

	Lung metastasis	Lymph node metastasis	*P* value (Fisher’s exact test)
LM1-S3	8/8	0/8	0.0002
LM1-S4	8/8	0/8	0.0002
LM1-S5	8/8	0/8	0.0002
LM1-S11	6/8	0/8	0.0070
LnM1-S11	8/8	8/8	—
LnM1-S13	2/8	2/8	1.000

Forty-eight nude mice were divided randomly into 6 groups with 8 ones in each group. Qualitative variables were compared using Fisher's exact test. Kruskal-Wallis was used to analyze qualitative variables among these six cell lines

**Table 2:** Metastatic tropisms of monoclonal cell lines gauged by fluorescence area (*pixel*)

	Lung metastasis area	Lymph node metastasis area	Z value	*P* value
LM1-S3	176820 ± 13120	0	−2.521	0.012
LM1-S4	1296000 ± 524228	0	−2.521	0.012
LM1-S5	34676 ± 7388	0	−2.521	0.012
LM1-S11	499011 ± 133568	0	−2.521	0.012
LnM1-S11	486529 ± 180184	884586 ± 442968	−1.820	0.069
LnM1-S13	41132 ± 23599	21873 ± 4712	−1.820	0.069
*χ* ^2^	40.71	46.39		
*P* value (Kruskal-Wallis)	<0.0001	<0.0001		

Pixel represented the fluorescence area. Wilcoxon rank test was used to test the same cell line metastasis variables. Kruskal-Wallis was used to analyze qualitative variables among these six cell lines

### Lung colonization of monoclonal HCC cell lines

Colonization and outgrowth at a distant site are the key final step in the process of metastatic cascade and, more importantly, colonization is the basis of organ specific metastasis. To test the relationship between colonizing ability and lung metastatic potential, the monoclonal cells were injected into the nude mice via tail vein. The dynamic changes of HCC cells in lung were observed using fluorescent signal in this tumor models. Shortly after injection, many HCC cells were non-specifically trapped in the lung, and then tumor cells were dramatically attenuated within the first few days due to blood perfusion and tumor apoptosis. At 72 h after injection, a significant difference in lung colonization of these monoclonal HCC cells was monitored (Fig. 2c). As expected, LM1-S4 exhibited the highest lung colonizing ability in all tested cells including its parental cells (*p* <0.001; Fig. 2c). Moreover, LnM1-S11 had the second highest capability of lung colonization in all tested cells (*p* <0.001; Fig. 2c).

### Proliferation, migration and invasion abilities of monoclonal HCC cells

**Fig. 3:**
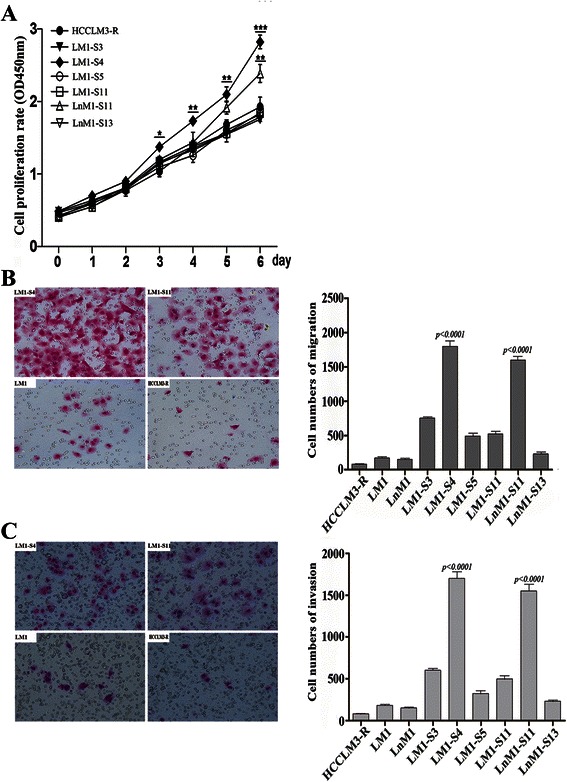
Proliferation, migration and invasion abilities of monoclonal HCC cells. **a** Cell proliferation curves of monoclonal HCC cells and their parental cells. **b** & **c** Representative images (*left*) and their statistical results (*right*) of monoclonal HCC cells using migration and invasion assays

The capabilities of tumor proliferation, migration and invasion are the basic important features to complete a whole process of tumor metastasis. Therefore, we compared cell proliferation of each monoclonal HCC cells to its parental cells in parallel. During a 7-days’ observation, LM1-S4 grew the fastest (*p* <0.0001) and LnM1-S11 grew the second fastest of all tested HCC cell lines (*p* <0.01; Fig. 3a). No significant differences in cell proliferation were found between LM1, LnM1, LM1-S3, LM1-S5, LM1-S11 and LM1-S13 cells.

Next, we assessed the migration and invasion abilities of these monoclonal HCC cell lines. First, we investigated the migration capability in LM1-S4, LM1-S11, LM1, and HCCLM3-R. The results showed that LM1-S4 had highest abilities of motility in all tested cell lines (*p* <0.0001; Fig. 3b). And LnM1-S11 exhibited stronger capabilities of migration than other cell lines, except of LM1-S4 (*p* <0.0001; Fig. 3b). Next, we investigated the invasion capability in LM1-S4, LM1-S11, LM1, and HCCLM3-R. The results showed that invasion capabilities of LM1-S4 and LnM1-S11 were much higher than other cell lines (*p* <0.0001; Fig. 3c). Collectively, our findings suggest that the metastatic potentials of these monoclonal cell lines were remarkably different.

### Tropism-related genes in HCC metastasis

**Fig. 4:**
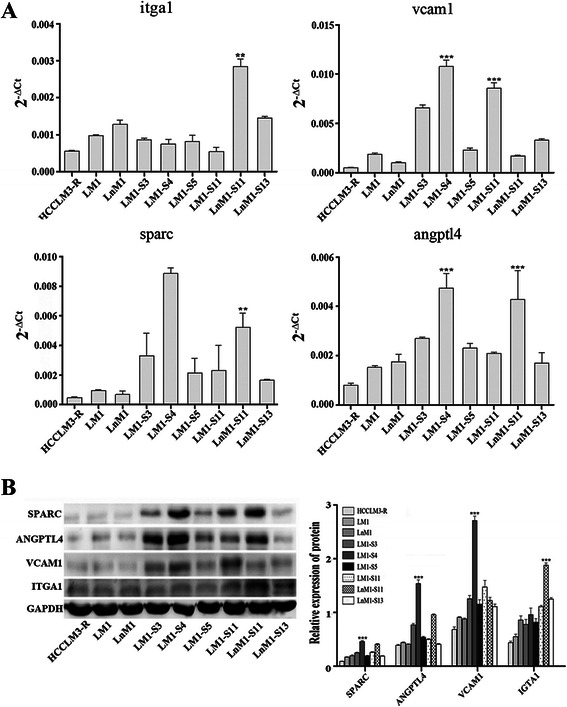
Tropism-related genes in HCC metastasis. **a** The endogenous mRNA levels of ITGA1, VCAM1, SPARC and ANGPTL4 in monoclonal HCC cells analyzed by Real-time PCR analysis. **b** The protein levels of ITGA1, VCAM1, SPARC and ANGPTL4 of monoclonal HCC cells quantified by Western blot. GAPDH was included as loading control. Data are presented as mean ± SEM, One-way ANOVA was used

**Fig. 5:**
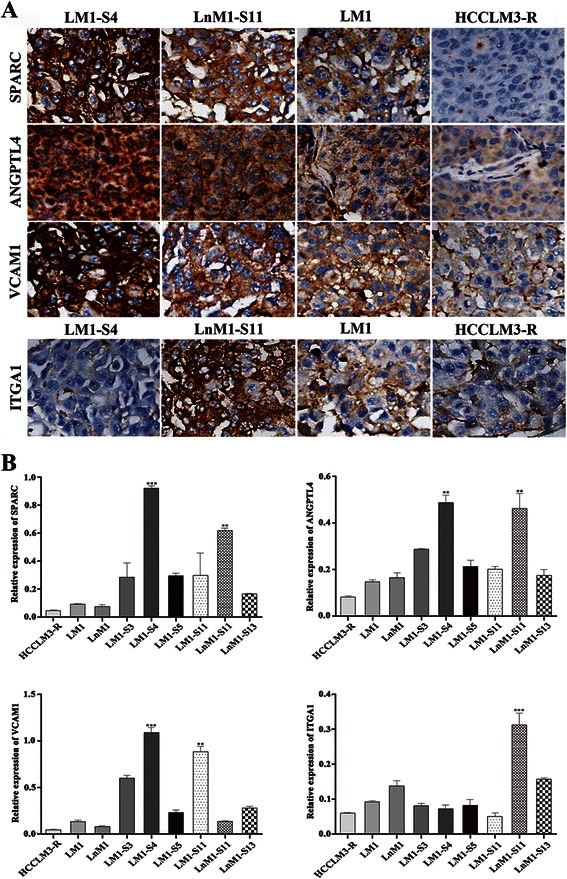
Protein levels of ITGA1, VCAM1, SPARC and ANGPTL4 in xenograft tissues. Representative images (**a**, ×100) and Quantitative results (**b**) of ITGA1, VCAM1, SPARC and ANGPTL4 protein levels in primary xenograft tissues using immunohistochemical staining analyses. Data are presented as mean ± SEM, One-way ANOVA was used

More and more evidences indicated that tumor metastatic potential and tropism were regulated by different panel of genes. To identify the key drivers of HCC metastatic tropisms, we first reviewed several recently published articles and selected 30 candidates which had been found to regulate tumor metastatic tropisms in breast, prostate and lung cancer, etc [19–25] (Additional file 1: Table S1). Then, the expression levels of these genes were analyzed by real-time PCR and only 21 genes were successfully detected in our subclonal cells (Additional file 2: Table S2 and Additional file 3: Figure S1). Four differentially expressed genes, named SPARC, ANGPTL4, VCAM1 and ITGA1, were finally picked out because their mRNA levels were more than 1.5-fold up-regulated in over half of monoclonal cell lines as compared with their parental cells (Fig. 4a). To validate above results, the corresponding proteins were detected again by Western blots in these monoclonal cell lines. Indeed, the protein levels of SPARC, ANGPTL4 and VCAM1 were remarkably correlated with the potentials of lung metastasis, while ITGA1 was positively related to celiac lymph node metastasis (*p* < 0.01; Fig. 4b). To analyze protein levels in vivo, tumor tissues from monoclonal HCC xenografts and their parental HCC xenograft were stained by immunohistochemistry. Once again, SPARC, ANGPTL4 and VCAM1 were highly expressed in both LM1-S4 and LnM1-S11 xenograft tissues, while ITGA1 only in LnM1-S11 xenograft tissues (Fig. 5a and b). All those results indicated that SPARC, ANGPTL4, VCAM1 and ITGA1 might be the key drivers of HCC metastatic tropisms.

## Discussion

Tumor metastasis has been a bottleneck in HCC prognosis and therapy. To elucidate the underlying mechanisms, metastatic human HCC cell lines and their corresponding xenograft models had been widely used to mimic the metastatic cascade of HCC in human being. For the purpose, a serial of human HCC cell lines, such as MHCC97, HCCLM3 and HCCLM6, with high metastatic potentials, were successfully established at Liver Cancer Institute in the past decade [10–12]. To monitor the real-time process of HCC metastasis in vivo, HCCLM3-R, a stably RFP expressed cell, was used for establishment of xenograft model [13]. Taking the advantage of red fluorescent signaling, multiple metastatic foci in liver, lung as well as lymph nodes of posterior peritoneum were easy to be visualized in nude mice as early as 2 weeks after orthotopic transplantation. The results obviously suggested that HCCLM3-R is a heterogeneous population with different metastatic tropisms.

As the heterogeneity of tumor cells would complicate to clarify the issue of HCC metastatic tropism, we decided to isolate monoclonal cells from HCCLM3-R. By the virtue of RFP signaling, two direct descendent HCC cell lines, named LM1 and LnM1, were successfully established from lung and lymph node metastatic foci of HCCLM3-R xenograft. By limiting dilution in vitro, more monoclonal cell lines, such as, LM1-S3, LM1-S4 and LM1-S11 were sequentially established from LM1 cells, and LnM-S11 and LnM-S13 from LnM1 cells as well. Still, the metastatic potentials and tropisms of these monoclonal cell lines were remarkably different. For example, the former 3 monoclonal cells had a propensity of lung metastasis, while the latter 2 cells exhibited dual metastatic tropisms. LM1-S4 showed the highest capability of lung metastasis, and LnM1-S11 had the highest dual-metastatic capabilities, both to lung and lymph node. By far, we successfully established a serial monoclonal HCC cell lines from one parental cell line in the world. These monoclonal HCC cell lines, together with their xenograft models would provide us a powerful tool for HCC tropism study.

Although there are a lot of hypotheses on tumor metastasis and metastatic tropism, none of them can explain well on all issues seen in metastatic cascade. Stephen Paget’s “seed and soil” theory, for example, is the most famous one where he postulated that metastases of a particular type of cancer ("the seed") often metastasizes to certain sites ("the soil") based on the similarity of the environments of the original and secondary tumor sites [5, 26]. Until now, the theory is still widely accepted and further explained by the concept of interplay between the tumor and its microenvironment. Meanwhile, the theory of Darwinian’s selection is also an active one, in which only a few clonal tumor cells is endowed with all abilities to complete the whole cascade of metastasis [27, 28]. Our observations in this study confirmed the right of Darwinian’s selection hypothesis once more, because only a few of isolated monoclonal HCC cells were able to successfully grow in vitro and even more few progressive metastasis in xenograft models. These preliminary results indicated that HCCLM3 was composed of different subsets with district proliferation, invasiveness, and metastatic tropisms.

In these 30 candidates, both mRNA and protein levels of SPARC, ANGPTL4 and VCAM1 were finally found significantly up-regulated in most monoclonal HCC cell lines with lung metastasis potential, especially in LM1-S4, as compared to their parental cells. And ITGA1 were mainly increased in LnM-S11, a monoclonal cell with high lymph node tropism. All these results indicated that SPARC, ANGPTL4 and VCAM1 might be a core panel of drivers modulating HCC lung metastasis, while ITGA1 is an important driver regulating HCC lymph node tropism. Although these observations are similar to the previous ones in melanoma, and breast cancer, in which SPARC is required in extracellular matrix synthesis [21]; ANGPTL4 weakens cell-cell conjunctions by initiating integrin α5β1-mediated RAC/PAK signaling [22]; VCAM1 often alters cell adhesion as a cell surface sialoglycoprotein [23]; and ITGA1 mediates cell-collagen or cell-laminin adhesion [24, 25]. Furthermore, the differential expression levels of IGTA1, VCAM1, SPARAC and ANGPTL4 in these subclonal cells have been further confirmed in our mRNA sequencing data (GEO accession: GSE38945). All these results suggest that IGTA1, VCAM1, SPARAC and ANGPTL4 could be the drivers of HCC metastatic tropisms. Although, the gains-of-functions or/and loss-of-functions of these candidates need be further explored [29, 30].

## Conclusions

In a word, several monoclonal HCC cell lines and their xenograft models were successfully established from HCCLM-R cells in this study. By these powerful in-vitro and in-vivo platforms, we believed, HCC metastatic tropisms in clinic could be easily mimicked and recapitulated for exploring the bilateral interactions between tumor and its microenvironment, elucidating the drivers of HCC metastatic tropisms, and testing anti-cancer effects of newly developed agent in pre-clinical stage.

## Additional files

Additional file 1: Table S1. Thirty candidate genes whose functions are related to tropism metastasis of breast, prostate and lung cancer, etc. were picked out from reference. (PDF 84 kb)
Additional file 2: Table S2. Primer sequences of qRT-PCR analyses for 21 genes. (DOCX 17 kb)
Additional file 3: Figure S1. Thirty candidate genes whose expression levels were analyzed by real-time PCR and 21 genes were successfully detected in our subclonal cells. (TIFF 9925 kb)

## Abbreviations

HCC: Hepatocellular carcinoma
IHC: Immunohistochemical staining
IOD: Integral optical density
SPARC: Secreted protein acidic
VCAM1: Vascular cell adhesion molecule 1
ANGPTL4: Angiopoietin-like 4
ITGA1: Integrin, alpha 1
