# Supplementary material for: Establishment of monoclonal HCC cell lines with organ site-specific tropisms
Source: BMC Cancer. 2015 Oct 12;15:678. doi: 10.1186/s12885-015-1692-0 (PMC4603809; doi:10.1186/s12885-015-1692-0)
Supplement: Additional file 1: Table S1. — Thirty candidate genes whose functions are related to tropism metastasis of breast, prostate and lung cancer, etc. were picked out from reference. (PDF 84 kb) [file 12885_2015_1692_MOESM1_ESM.pdf]

gene	lung meastasis	lymph node metastasis	typer-of-tumor	author	title	source
NY-BR-1		√	breast cancer	Varga Z	Preferential Nuclear and Cytoplasmic NY-BR-1Protein Expression in Primary Breast Cancer and Lymph NodeMetastases	Clin Cancer Res 2006;12(9):2745
VEGF-C/D,		√	breast cancer	Stacker SA	VEGF-D promotes the metastatic spread of tumor cells via the lymphatics.	Nat Med 2001,7:186–191
				Skobe M	Induction of tumor lymphangiogenesis by VEGF-C promotes breast cancer metastasis.	Nat Med 2001,7:192–198
(DSC2/UGT8/ITGB8/FERMT1/TFCP2L1/ANP3	√		breast cancer	Landemaine T	A Six-Gene Signature Predicting Breast Cancer Lung Metastasis	Cancer Res 2008;68(15):6092-9
IL11/CTGF/CXCR4			breast cancer	Minn AJ	Distinct organ-specific metastatic potential of individual breast cancer cells and primary tumors	*J. Clin. Invest.* 2005, 115:44–55.
MMP1	√					
LAMC2		√	bladder cancer	Smith SC	Profiling Bladder Cancer Organ Site-Specific Metastasis Identifies LAMC2 as a Novel Biomarker of Hematogenous Dissemination	Am J Pathol 2009, 174:371–379;
EpCAM	√					
αv（CD51）β3（CD61） integrins				Kischel P	Cell Membrane Proteomic Analysis Identifies Proteins Differentially Expressed in Osteotropic Human Breast Cancer Cells	Neoplasia 2008，10：1014–1020
Prohibitin						
MRP-1						
cyclooxygenase COX2	√		breast cancer	Bos PD	Genes that mediate breast cancer metastasis to the brain	Nature 2009; 459(7249): 1005–1009.
HBEGF	√					
α2,6-sialyltransferase ST6GALNAC5						
ANGPTL4	√					
latent TGF-β-binding protein (LTBP1）	√					
fascin-1 (FSCN1)	√					
SPARC	√		breast cancer	Lu X	Efficient acquisition of dual metastasis organotropism to bone and lung through stable spontaneous fusion between MDA-MB-231 variant	PNAS 2009，106（23）:9385–9390
IL13RA2	√					
VCAM1	√					
Id	√					
MMP1	√					
IL11						
CXCR4						
ADAMTS1						
MCAM						
CTGF						
PTEN			prostatic cancer/bladder cance	Wu Z	The role of PTEN in prostate cancer cell tropism to the bone micro-environment	Carcinogenesis 2007， 28（7）:1393–1400
CD73		√	breast cancer	Lee H	GENE EXPRESSION PROFILING OF TUMOR XENOGRAFTS:*IN VIVO* ANALYSIS OF ORGAN-SPECIFIC METASTASIS	Int J Cancer 2003， 107，528–534
Integrin, alpha 1 H68922 3.8		√				
Pleiotrophin		√				
αv-integrin			prostatic cancer	van der Horst G	Targeting of αv-Integrins in Stem/Progenitor Cells and Supportive Microenvironment Impairs Bone Metastasis in Human Prostate Cance	Neoplasia 2011 13, 516–525
