# Supplementary material for: Establishment of monoclonal HCC cell lines with organ site-specific tropisms
Source: BMC Cancer. 2015 Oct 12;15:678. doi: 10.1186/s12885-015-1692-0 (PMC4603809; doi:10.1186/s12885-015-1692-0)
Supplement: Additional file 2: Table S2. — Primer sequences of qRT-PCR analyses for 21 genes. (DOCX 17 kb) [file 12885_2015_1692_MOESM2_ESM.docx]

**Supporting information Table 1** Primer sequences for qRT-PCR analyses in this study

| desmocollin 2-F | ACACGGCCCAAAACTATACCA |
| --- | --- |
| desmocollin 2-R | CTCACGATCTACAGGACGAGTA |
| UGT8-F | TGACAGCAATCGAACTGTTTGA |
| UGT8-R | CCACACATATCATTAGGGTCCAC |
| ITGB8-F | ACCAGGAGAAGTGTCTATCCAG |
| ITGB8-R | CCAAGACGAAAGTCACGGGA |
| FERMT1-F | GCGTTGACCATCCCAATGAAG |
| FERMT1-R | ACCAAAGAGCAAAGTCTGACC |
| TFCP2L1-F | CGTTTAAGCAGAACGAGAATGGG |
| TFCP2L1-R | TTTCATAGGACGGCTGGTATTTC |
| ANP32E-F | TGCCTGTGTGTCAATGGGG |
| ANP32E-R | GCAGAGCTTCTACTGTACTGAGA |
| LAMC2-F | GACAAACTGGTAATGGATTCCGC |
| LAMC2-R | TTCTCTGTGCCGGTAAAAGCC |
| EpCAM-F | ATCGTCAATGCCAGTGTA |
| EpCAM-R | CTGCCTTCATCACCAAAC |
| COX2-F | CGAGGTGTATGTATGAGTGT |
| COX2-R | AGTGGGTAAGTATGTAGTGC |
| HBEGF-F | ATCGTGGGGCTTCTCATGTTT |
| HBEGF-R | TTAGTCATGCCCAACTTCACTTT |
| ANGPTL4-F | GGCTCAGTGGACTTCAACCG |
| ANGPTL4-R | CCGTGATGCTATGCACCTTCT |
| IL-8-F | CTGACGGCCACGAACTTCC |
| IL-8-R | GCACTGACATTTGTCCCTTGA |
| LRBP1-F | TCCCGACTGCCGTTTCCT |
| LRBP1-R | TGACGCTGTAGATGTTGACCTG |
| SPARC -F | AGGAAACCGAAGAGGAGG |
| SPARC -R | TTGTGGCAAAGAAGTGGC |
| IL13RA2-F | GGGCATTGAAGCGAAGATACA |
| IL13RA2-R | GCCCAGGAACTTTGAACTTCTG |
| VCAM1-F | GGGAAGATGGTCGTGATCCTT |
| VCAM1-R | TCTGGGGTGGTCTCGATTTTA |
| ID1-F | CTGCTCTACGACATGAACGG |
| ID1-R | GAAGGTCCCTGATGTAGTCGAT |
| MMP1-F | GGGGCTTTGATGTACCCTAGC |
| MMP1-R | TGTCACACGCTTTTGGGGTTT |
| CD73-F | AAGGACTGATCGAGCCACTC |
| CD73-R | GGAAGTGTATCCAACGATTCCCA |
| ITGA1-F | CTGGACATAGTCATAGTGCTGGA |
| ITGA1-R | ACCTGTGTCTGTTTAGGACCA |
| PTN-F | CCTGCACAATGCCGAATG |
| PTN-R | ATCCAGCATCTTCTCCTGTTTC |
| β-Acitin-F | CACCATGAAGATCAAGATCATTGC |
| β-Acitin-R | GGCCGGACTCATCGTACTCCTGC |
