# Supplementary figures and images for: Establishment of monoclonal HCC cell lines with organ site-specific tropisms
Source: BMC Cancer. 2015 Oct 12;15:678. doi: 10.1186/s12885-015-1692-0 (PMC4603809; doi:10.1186/s12885-015-1692-0)

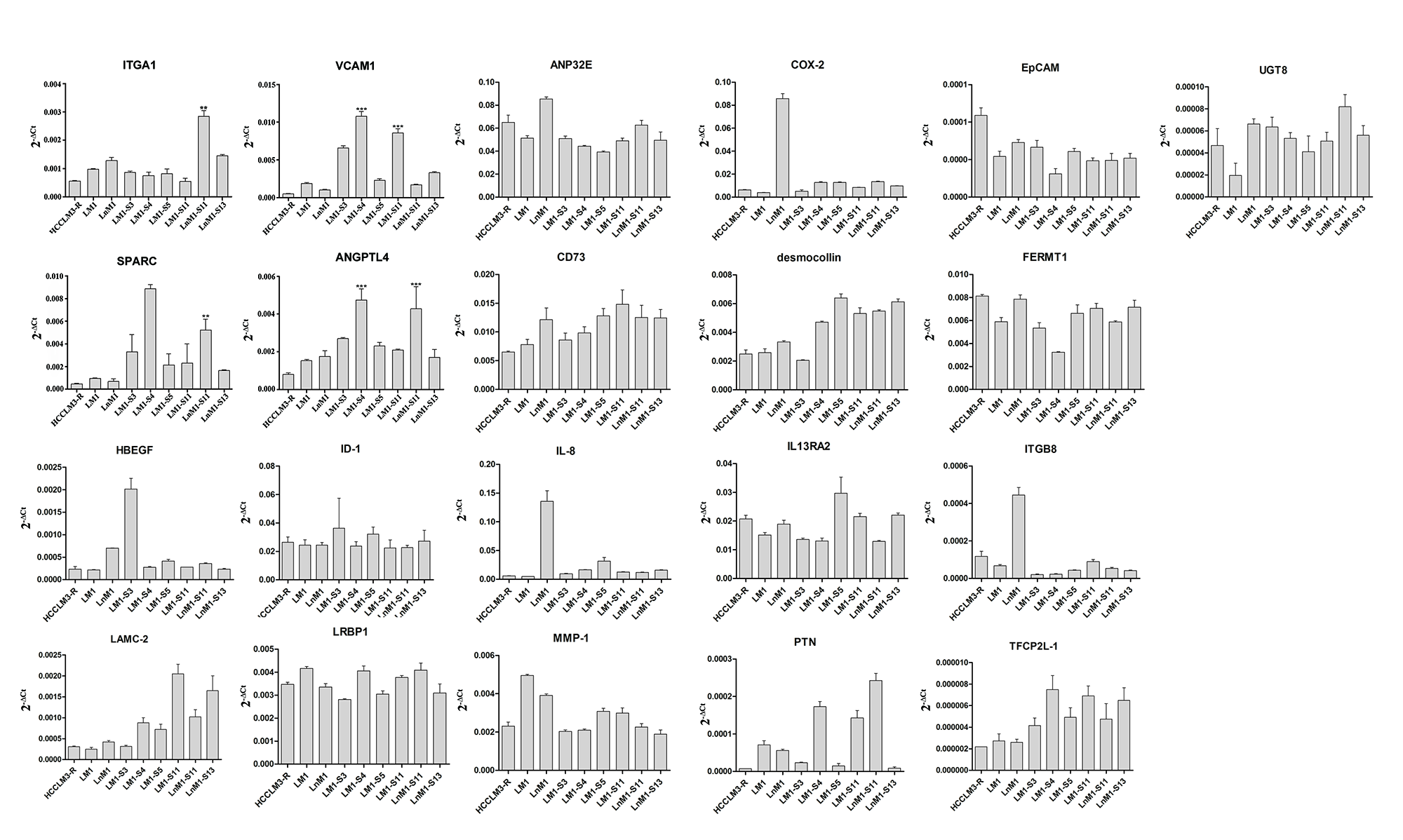

Supplement: Additional file 3: Figure S1. — Thirty candidate genes whose expression levels were analyzed by real-time PCR and 21 genes were successfully detected in our subclonal cells. (TIFF 9925 kb) [file 12885_2015_1692_MOESM3_ESM.tiff]
